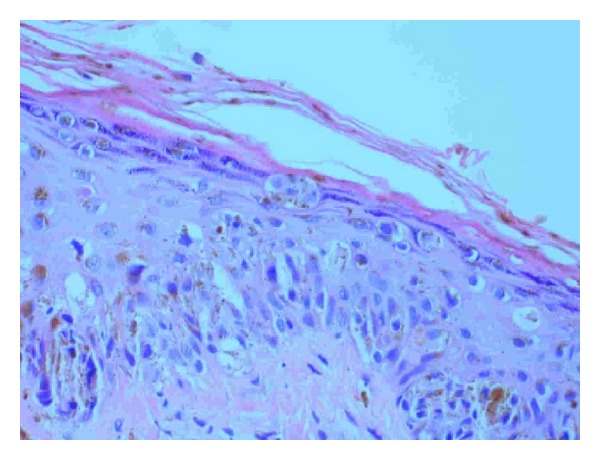# Micromelanomas: A Review of Melanomas **≤**2 mm and a Case Report

**DOI:** 10.1155/2014/206260

**Published:** 2014-01-19

**Authors:** Sharad P. Paul

**Affiliations:** ^1^Skin Surgery Clinic, 271A Blockhouse Bay Road, Auckland, NZ 0600, New Zealand; ^2^Department of Skin Cancer, School of Medicine, University of Queensland, Qld 4029, Australia; ^3^Faculty of Surgery, University of Auckland, NZ 1142, New Zealand

## Abstract

The ABCD acronym used to screen pigmented lesions for melanoma obviously was not designed to contend with melanomas that are under 2 mm in diameter. Previously, views ranged that such small lesions could not be melanomas until a few reports of such “micromelanomas” emerged. The author presents a 2 mm melanoma in situ presenting as an insignificant pigmented lesion in a 60-year-old patient with no previous history of melanoma or multiple nevi—which is usually the norm in cases of small melanoma. This paper reiterates the fact that when it comes to a melanoma, size does not matter. In this paper, the term “micromelanoma” is used by the author to represent melanomas under 2 mm. Dermatoscopy and histopathology findings are discussed in this case, along with a review of small melanomas.

## 1. Background

In 1985, the oft-quoted ABCD acronym was developed for melanoma screening as a public health tool to aid the diagnosis of melanomas [1]. Asymmetry, border, color, and diameter of the pigmented lesion were parameters discussed in this context. In 1987, Schmoeckel and Braun-Falco even suggested that pigmented lesions under 5 mm cannot be considered melanomas as clinical and histological features only became apparent when lesions enlarged beyond 5 mm size [2]. Then a study from the Sydney Melanoma Unit undertook a large retrospective study and concluded that 31.1% of lesions were 6 mm or less in diameter [3]. After adjusting for tissue shrinkage among specimens from this Australian cohort, it was reported that only 10% of invasive melanomas were small-diameter tumors [4]. A few years later, a paper presented a series of invasive small-diameter melanomas, debating if the “D” should be removed from the ABCD acronym [4]. Recently, a case report reviewed the dermatoscopy and dermatopathology findings of a tiny invasive melanoma in a 38-year-old patient who had >100 nevi—with the smallest diameter ever of a reported melanoma of 1.6 mm [5]. Some groups reported that small-diameter melanoma tumor thicknesses ranged from 0.11 to 1.5 mm, with a median thickness of approximately 0.7 mm [6].

The author presents a 2 mm melanoma in situ presenting as a solitary de novo lesion in a 60-year-old patient with no previous history of melanoma or multiple nevi—illustrating the fact that when it comes to a melanoma, established clinical patterns or size do not seem to matter. As many authors have already stated, the ABCD criteria often does not seem to matter [7, 8]. In this paper, the term “micromelanoma” is coined to represent melanomas under 2 mm.

The presentation is also unusual because of the age and clinical presentation of this lesion not being clinically different to the patient's other nevi. Further this patient had <5 nevi overall. This lesion did not look particularly sinister on clinical examination with the naked eye. The dermatoscopic and histological aspects are reviewed in the context of this clinical case and the associated literature of “micromelanomas.”

## 2. Case History

**Figure 1:**
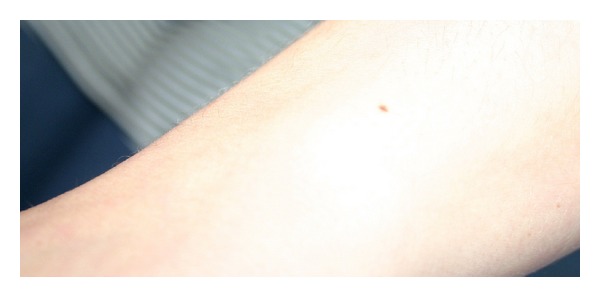


A 60-year-old lady (Caucasian, Fitzpatrick Type 2 skin) presented for a screening skin examination with no previous family history or significant personal medical history of skin cancer. On examination she had a very small 2 mm pigmented lesion on her right forearm (Figure 1). She had not been aware of this lesion given its tiny size. She had few nevi (<5) and all other nevi appeared equally pigmented and around 2 mm in diameter. None of them appeared particularly dark on clinical examination.

## 3. Dermatoscopy

**Figure 2:**
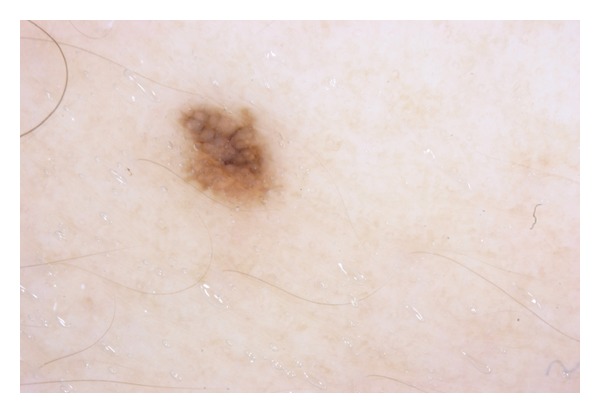


Dermatoscopy has now become well established as a technique to detect early melanomas or to screen pigmented lesions. On examination with a dermatoscope (Heine Delta 20 dermatoscope, manufactured by Heine, Optotechnic GmbH, Herrsching, Germany), the lesion being discussed had no obvious melanin network, but it had asymmetry of color; further, the blueness suggested that is was probably both melanocytic and atypical (Figure 2). Small melanomas are not only missed by the ABCD rule, but dermatoscopy is notoriously difficult, with most dermatoscopic algorithms not being useful [9].

Looking for “Chaos and Clues” in dermatoscopy has been described as an extremely useful method [10]. In this method “chaos” is defined as the presence of asymmetry in structure or color. In the presence of “chaos” one looks for any of the following eight clues:

1. thick reticular lines,
2. grey or blue structures of any kind,
3. pseudopods or radial lines at the periphery,
4. black dots in the periphery,
5. eccentric structure-less area of any color,
6. polymorphous vascular pattern,
7. white lines,
8. parallel lines on ridges.

In the case being described here, the lesion exhibited “chaos” (asymmetry of color or structure) and also a “clue” (grey or blue structure of any kind). Therefore, excision biopsy was done. Interestingly, this lady had very few (<5) nevi and none of the other equally small and pigmented nevi exhibited any asymmetry.

In dermatoscopy, most two-step algorithms commonly recommended were established to differentiate melanocytic from nonmelanocytic lesions as a first step. However, using a “chaos and clues” method helps us differentiate malignant from benign lesions first—by looking for chaos over symmetry. In comparing these methods, Tschandl et al. and others commented that looking for chaos and clues was preferable—given that the first step of the traditional dermatoscopic 2-step algorithm, if applied consistently, has a low specificity especially in patients with severely sun-damaged skin, as is often found in Australasia [11].

## 4. Histopathology

Ferrara et al. suggest that small melanomas need more stringent criteria and a “consensus approach” to diagnosis among examining pathologists, as there is no gold standard. In their study they suggest that severe cytologic atypia represents a useful clue in differentiating small melanomas from small dysplastic nevi [12].

**Figure 3:**
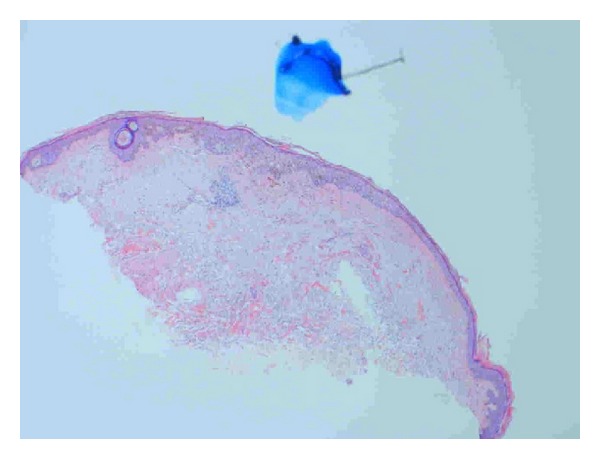


**Figure 4:**
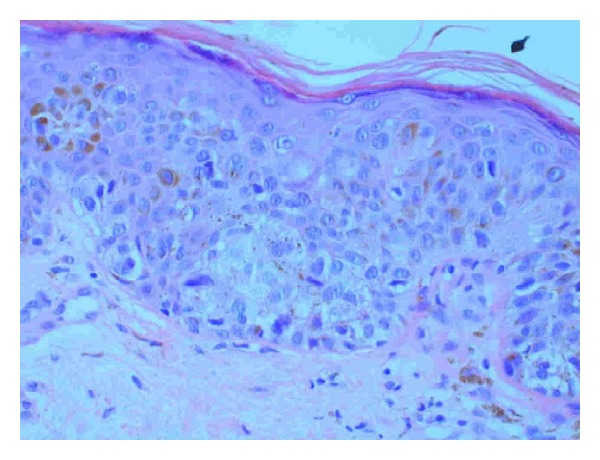


Sections here show superficial sun-damaged skin bearing a small proliferation of atypical melanocytes showing pagetoid scatter to the granular layer along with trans-epidermal elimination of melanin pigment. Superficial dermis shows melanophages and there is no dermal invasion. The appearance is suggestive of a melanoma in situ because of the combination of cytologic atypia and epidermal invasion (Figures 3, 4, and 5).  Figure 3 shows the biopsy specimen; Figure 4 shows atypical hyperchromatic melanocytes singly and in nests; and Figure 5 shows transepidermal (pagetoid) invasion.

## 5. Discussion

Micromelanomas (used by the author to denote melanomas under 2 mm diameter) as discussed earlier naturally will not fit the ABCD acronym. In earlier studies of pigmented lesions 3 to 6 mm in diameter, authors showed that clinical criteria for diagnosing melanoma are not as reliable in the case of pigmented lesions less than 6 mm diameter [13]. In this case of a tiny pigmented lesion of 2 mm diameter, the unusual features were the absence of several nevi, which is usually the case in other reported cases. In this situation, this was the only nevus that exhibited any chaos and therefore using the “chaos and clues” algorithm proved decisive.

The lesion turned out to be a melanoma in situ and was managed by wide surgical excision to ensure 5 mm margins all around. There were no complications or further issues to do with this lesion.

## 6. Conclusion

Micromelanomas, melanomas under 2 mm, are being increasingly reported and given the minute size, the ABCD screening acronym becomes redundant. Further, traditional two-dermatoscopic diagnostic methods often fail and the “chaos and clues” algorithm may be the better method to follow while performing dermatoscopy. In previously reported micromelanomas, the lesions were noted to be darker than other nevi (the so-called “ugly duckling” sign) [14]. Further, patients usually had dysplastic nevus syndrome with >100 nevi. Our patient exhibited neither of the above clinical features. The lesion was one of 4 nevi and all appeared similar to the naked eye and not particularly abnormal. However, when all the nevi were examined using a dermatoscope, this particular lesion proved significant when using the “chaos and clues” method of dermatoscopy; histology confirmed features of a melanoma in situ. Therefore, this case further serves to reinforce that when it comes to melanoma or melanoma in situ, size or number of nevi does not matter. Further, using dermatoscopy one is able to diagnose these “micromelanomas” at a very early stage of evolution—both from a histological and dimensional point of view.

## Figures and Tables

**Figure 5:**